# Characterizing Veteran Engagement Groups for Research at the US Department of Veterans Affairs

**DOI:** 10.2196/108688

**Published:** 2026-10-23

**Authors:** Anna V Kasdan, Bonnie J BootsMiller, Kristina M Cordasco

**Affiliations:** 1 Office of Research and Development, United States Department of Veterans Affairs, 810 Vermont Ave NW, Washington, DC, 20420, United States; 2 Science & Technology Policy Fellowship, American Association for the Advancement of Science, Washington, DC, United States; 3 Center for the Study of Healthcare Innovation, Implementation, and Policy, VA Greater Los Angeles Healthcare System, Los Angeles, CA, United States; 4 Department of Medicine, University of California, Los Angeles, Los Angeles, CA, United States

**Keywords:** Veterans, research engagement, health systems research, participatory methods

## Abstract

At the US Department of Veterans Affairs (VA), Veteran engagement groups (VEGs)—an integral component of VA research—have two-way impacts, as reported by group facilitators: they ensure that VA researchers, clinicians, and operational leaders conduct Veteran-centric work, and they confer several benefits, including health and social benefits, to Veteran members.

## Introduction

Patient engagement groups in research have been shown to improve the feasibility and quality of research while simultaneously providing patients with a voice in improving health and health care for themselves and others [1,2]. At the US Department of Veterans Affairs (VA), the Health Systems Research (HSR) portfolio in the Office of Research and Development (ORD) has a strong history of involving Veterans in all aspects of the research process through Veteran engagement groups (VEGs) [3].

Prior work on subsets of HSR VEGs highlights that although local implementation varies by research center location, groups share a common goal of providing Veterans with opportunities to shape the research that is for and about them [4-6]. To assess the state of the field and better understand the scope of VEG efforts, we characterized VEGs affiliated with all VA HSR centers as of 2025, focusing on their structure, composition, activities, and impacts.

## Methods

### Overview

A brief survey was developed in VA Research Electronic Data Capture (REDCap; version 14.12.0) and distributed via email by ORD staff (AVK, BJB) to VEG facilitators across all VA HSR Centers of Innovation (COINs; n=20) and Consortia of Research (COREs; n=7) in May 2025. We used closed-ended questions to assess VEG geographic location, group size, member makeup, and meeting frequency and format. Open-ended questions were used to assess topic foci, activities, and impacts. Respondents could also optionally share anonymous quotes from Veterans about the impacts of participating in VEGs. In the initial email, respondents were informed that responses would not influence ORD’s support for their VEG.

We generated descriptive statistics for closed-ended survey questions (analyses in RStudio, version 2024.12.1; Posit Software, PBC) and qualitatively analyzed open-ended survey responses using a constant comparison process [7]. Two authors (AVK, KMC) met via video conference on two separate days to identify and formulate consensus about themes for the questions about both VEG activities and impacts.

### Ethical Considerations

VA’s Central Institutional Review Board determined that project activities were quality improvement work and not research involving human subjects.

## Results

All VEG facilitators (100%) responded, providing information on 37 unique VEGs; some COINs/COREs had more than one affiliated VEG. All VEGs had ORD sponsorship and had significantly interacted with VA HSR research studies. About half of the VEGs (n=21) reported a specialty focus (eg, women Veterans, Veterans experiencing homelessness), while the remainder were more general in the research topics discussed. Groups ranged in size (3‐17 members, median: 9.5, IQR 8-12); 11 groups included caregivers in addition to Veterans, and 4 included VA employees or others. See Table 1 for additional VEG characteristics.

**Table 1.** Veteran engagement group characteristics.

Characteristics	Groups (N=37), n (%)
Member geography	
National	15 (40)
Local	15 (40)
Local, with some remote members	7 (19)
Meeting format	
Virtual	30 (81)
Hybrid	7 (19)
Meeting frequency	
Biweekly	2 (5)
Monthly	12 (32)
Bimonthly	8 (22)
Quarterly	7 (19)
Semiannually	4 (11)
Other	4 (11)

Responses to open-ended questions revealed that VEGs participate in activities along the full research lifecycle (Figure 1), with the goal of fostering more Veteran-centric research. Several VEGs noted that they also have social activities for rapport building, and some engage in strategic planning for their own VEG.

Veterans reported experiencing positive impacts from participating in VEGs, as relayed by VEG facilitators. These included making a difference and having their voices heard, having increased trust in VA research and clinical care, experiencing health and social benefits (eg, community building) and personal development (eg, seeking employment opportunities at VA), participating as members of research project teams and in high-profile research activities, and gaining new content knowledge on health topics and research processes.

**Figure 1.**
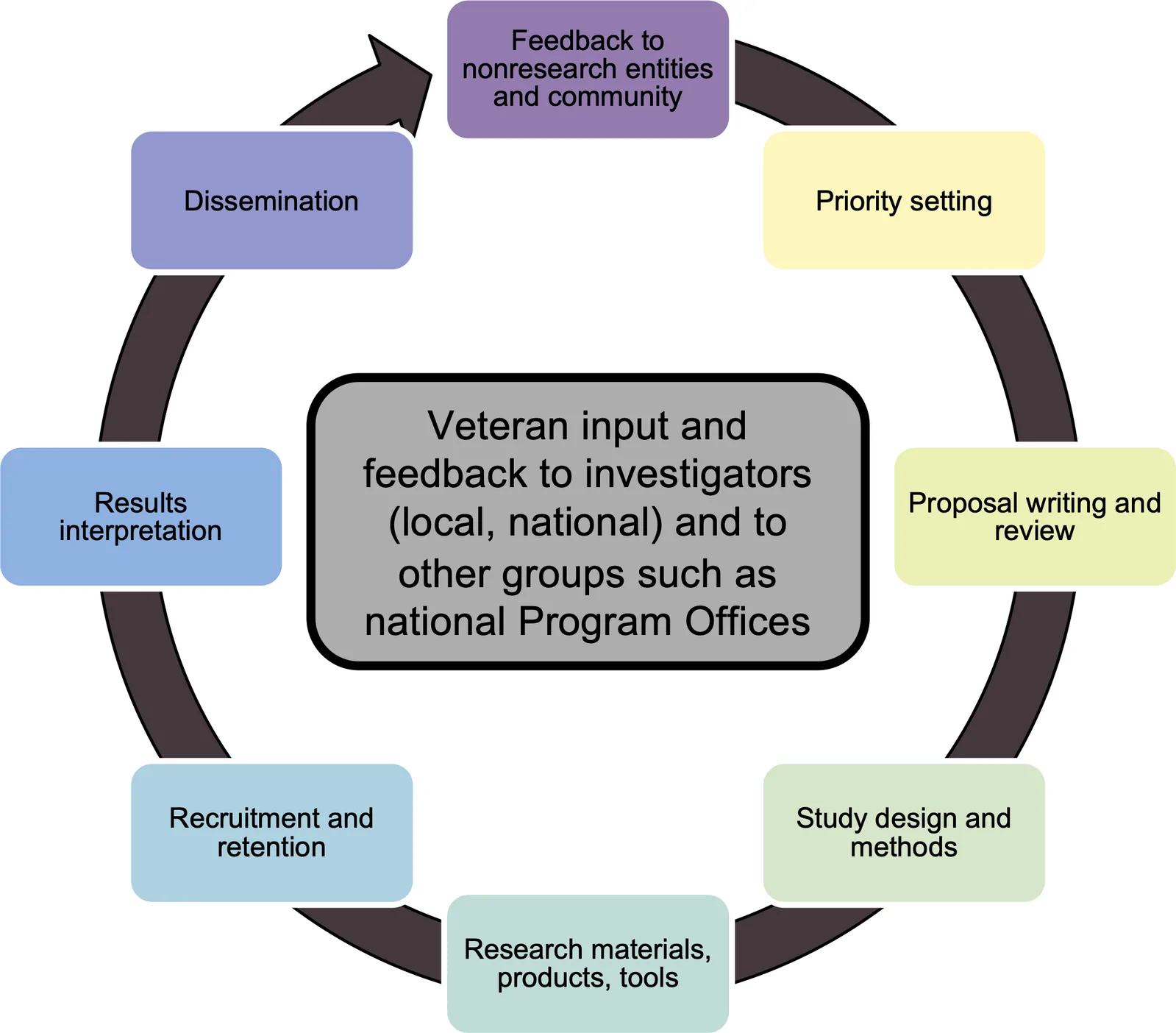
Summary of the full spectrum of the research lifecycle, which Veteran engagement group (VEG) members provide input on. Veterans’ health and care experiences are relevant for all aspects of the research lifecycle. Data are based on responses from 37 VEGs from the qualitative constant comparison analysis of VEG activities and impacts.

## Discussion

Veteran engagement groups (VEGs) connected with VA ORD’s HSR portfolio have bidirectional impacts: they ensure that VA researchers, clinicians, and operational leaders conduct work that stays Veteran centered, and these groups confer multiple benefits, including health and personal benefits, to Veteran members. VEGs vary in their specific structure and member composition, highlighting that they are a flexible mechanism for ensuring that research incorporates the unique perspectives and experiences of Veterans and their caregivers. Our assessment reinforces the benefits of VEGs, which have been described for subsets of these groups at the VA [6,8] and other patient research engagement groups [2], extending these findings to ORD’s HSR VEGs as a whole.

Patient participation and feedback are critical for the functioning of learning health care systems [9]. VA HSR’s engagement of Veterans through VEGs is a model that could be replicated by others for meaningfully engaging patients in large, complex organizations. In VA, this engagement model has led to measurable advances in care delivery by ensuring that Veteran priorities drive research priorities and thereby evidence-based system improvement [10].

One limitation of this work is that, due to our rapid assessment strategy, there may have been VEGs interacting with ORD HSR projects that were not identified (eg, rural groups consulting with HSR researchers that receive some ORD support). We also did not assess project-specific Veteran engagement efforts. Future work should focus on characterizing all VEGs associated with ORD, including those outside the HSR portfolio, to more comprehensively characterize the contributions Veterans make to all VA research areas. Second, our assessment did not systematically capture a few areas, including how engagement groups were set up, who VEGs provide feedback to (eg, only local VA researchers, any VA researcher nationwide, clinical operations partners), researcher demand for these groups, or how engagement is measured and tracked in projects. These gaps should be addressed in future work to better facilitate targeted support for VEG infrastructure and growth. Lastly, our work only captured the perspectives of VEG facilitators, rather than those of Veterans directly, which likely influenced the reporting of positive impacts. Future work should engage Veterans directly.

Overall, our work describes the current state of the field for HSR research engagement efforts, which can be extended to patient engagement groups more broadly. Given the meaningful outcomes for researchers and Veterans that come from participating in VEGs [6], there should be increased focus on prioritizing engagement efforts; one example already in place is ORD’s requirement for submitted research proposals to include a Veteran and provider engagement plan. Continued investment and infrastructure support efforts by ORD will optimize the impacts of VEGs, which directly advance VA’s mission to provide the best health care to Veterans.

## Abbreviations

COIN: Center of Innovation
CORE: Consortium of Research
HSR: Health Systems Research
ORD: Office of Research and Development
VA: Department of Veterans Affairs
VEG: Veteran engagement group
